# Easy-Scalable Flexible Sensors Made of Carbon Nanotube-Doped Polydimethylsiloxane: Analysis of Manufacturing Conditions and Proof of Concept

**DOI:** 10.3390/s22145147

**Published:** 2022-07-08

**Authors:** Antonio del Bosque, Xoan F. Sánchez-Romate, María Sánchez, Alejandro Ureña

**Affiliations:** Materials Science and Engineering Area, Escuela Superior de Ciencias Experimentales y Tecnología, Universidad Rey Juan Carlos, C/Tulipán s/n, Móstoles, 28933 Madrid, Spain; xoan.fernandez.sanchezromate@urjc.es (X.F.S.-R.); maria.sanchez@urjc.es (M.S.); alejandro.urena@urjc.es (A.U.)

**Keywords:** carbon nanotubes, stretchable sensors, PDMS, human motion monitoring

## Abstract

Carbon nanotube (CNT) reinforced polydimethylsiloxane (PDMS) easy-scalable sensors for human motion monitoring are proposed. First, the analysis of the dispersion procedure of nanoparticles into the polymer matrix shows that the ultrasonication (US) technique provides a higher electrical sensitivity in comparison to three-roll milling (3RM) due to the higher homogeneity of the CNT distribution induced by the cavitation forces. Furthermore, the gauge factor (GF) calculated from tensile tests decreases with increasing the CNT content, as the interparticle distance between CNTs is reduced and, thus, the contribution of the tunnelling mechanisms diminishes. Therefore, the optimum conditions were set at 0.4 CNT wt.% dispersed by US procedure, providing a GF of approximately 37 for large strains. The electrical response under cycling load was tested at 2%, 5%, and 10% strain level, indicating a high robustness of the developed sensors. Thus, this strain sensor is in a privileged position with respect to the state-of-the-art, considering all the characteristics that this type of sensor must accomplish: high GF, high flexibility, high reproducibility, easy manufacturing, and friendly operation. Finally, a proof-of-concept of human motion monitoring by placing a sensor for elbow and finger movements is carried out. The electrical resistance was found to increase, as expected, with the bending angle and it is totally recovered after stretching, indicating that there is no prevalent damage and highlighting the huge robustness and applicability of the proposed materials as wearable sensors.

## 1. Introduction

Nowadays, there is an increasing interest in the development of proper techniques for Structural Health Monitoring (SHM) applications, as it allows a continuous inspection of service structures, reducing the preventive maintenance and the costs that are associated to it. In this regard, there are a lot SHM techniques such as Fibre Bragg Grating (FBG) sensors, guided ultrasonic waves, or acoustic emission. In most of these cases, complex analytical and numerical tools are required and sometimes do not offer accurate information on the overall performance of the material [1,2]. For these reasons, it is necessary to explore other options.

Carbon nanoparticles and, most specifically, carbon nanotubes (CNTs) are an interesting alternative option for SHM purposes. The basis for SHM purposes lies in the fact that these conductive nanoparticles, when added into an insulating material such as a polymer to constitute a nanocomposite, promote the creation of an electrical percolation network, leading to a drastic enhancement of the electrical conductivity of the material. This electrical network will be affected by the presence of cracks or strain, which will be reflected in changes in the overall electrical conductivity due to the creation or breakage of conductive pathways [3,4,5,6]. Therefore, the application of carbon nanoparticles is now of great interest for damage detection in structural components. In this regard, some techniques such as Electrical Impedance Tomography (EIT) have gained much impact since they allow the damage to be easily detected, located, and quantified [7,8].

Apart from the use of carbon nanoparticles for SHM of structural components, their application for the development of strain sensors is now being extensively investigated [9,10,11,12]. This application is based on taking advantage of the alteration of the electrical percolation networks that occurs when the material is subjected to a deformation field that generates changes in the distances between neighboring nanoparticles. These changes have a very sensitive effect on tunnel conduction mechanisms, causing changes in the overall electrical performance of the whole material. More specifically, it is widely known that the electrical resistance associated to the tunnelling effect, also called tunnelling resistance, follows a linear-exponential correlation with the applied strain. This makes this type of sensor based on conductive nanoparticles very sensitive to mechanical deformation.

Within this group of materials, there is great interest in researching and developing wearable sensors based on CNT-reinforced silicon elastomers. These sensors must accomplish the following requirements: high values of failure strain, high sensitivity, high reproducibility, easy to manufacture, and user-friendly operation. Thereby, different silicone elastomers such as homemade silicone [13], Ecoflex [14,15,16,17], or PDMS [18,19,20,21,22,23,24,25,26,27] are being investigated to developed strain sensors. Here, it is essential to determine the adequate CNT content and reach the proper nanoparticle dispersion into the elastomer, as the CNT distribution plays a crucial role in the formation of a sensile electric percolation network which defines the final electromechanical properties of the sensor [28]. However, difficulties related to electrode placement on the surface of silicone nanocomposites remain to be investigated, due to the hydrophobic nature. In view of the state of the art, a strain sensor that achieves all the requirements has not yet been developed, since either they do not have a high sensitivity or flexibility, or a complex and expensive manufacturing method must be followed (for example CVD or laser ablation), or they are not user-friendly.

Thus, this study aims to develop a CNT-PDMS wearable sensor for human motion monitoring that meets the main requirements discussed above. Here, a study of the influence of dispersion procedure on the electrical and electromechanical properties was carried out to select the optimum manufacturing conditions to achieve the maximum sensitivity. In this regard, two simple dispersion techniques were evaluated: three-roll milling and ultrasonication. Finally, a proof-of-concept of human motion monitoring was conducted, proving the applicability of the proposed materials as wearable skin-mounted sensors.

## 2. Materials and Methods

### 2.1. Materials

Nanocomposites were manufactured with CNTs embedded in a silicone elastomeric matrix. The silicone elastomer was based on polydimethylsiloxane (PDMS) with the commercial denomination of SYLGARD 184TM, purchased from Dow^®^. The base monomer, with a viscosity of 5100 mPa·s, and the curing agent, with a viscosity of 3500 mPa·s, of PDMS were mixed in a 10:1 mass ratio. Multi-Wall Carbon Nanotubes (MWCNTs) used for this investigation were NC7000, purchased from Nanocyl^®^. They have an average diameter of 9.5 nm, a surface area of 250–300 m^2^/g, an average length of 1.5 μm, and a bulk density (EN DIN 60) of 66 kg/m^3^ with a 90% carbon purity.

### 2.2. Manufacturing of CNT-Doped PDMS Sensors

PDMS-based nanocomposites reinforced with CNTs were manufactured with a combination of two typical nanoparticle dispersion procedures in polymer matrices: three roll milling (3RM) and ultrasonication (US). CNT contents (0.25, 0.3, 0.4, 0.5, 0.75, and 1 wt.%) were selected to determine the percolation threshold of the CNT-PDMS system, that is, the critical CNT content in which it becomes electrically conductive; as well as, to explore the influence of the dispersion procedure on the electromechanical properties of the final nanocomposite. 

On one hand, CNTs were dispersed by 3RM process using an EXAKT 80E three roll milling machine. This technique consists of a progressive reduction of the gaps between consecutive rolls rotating at different speed ratios of 9:3:1 at room temperature, being 250 rpm the rotating speed of the fastest roll. The high shear forces induced by the rolls on the mixture promote a disaggregation of CNTs agglomerates. The 3RM process parameters were optimized in a previous study [29], and they are shown in Table 1.

On the other hand, CNTs were dispersed by US process using a HIELSCHER ULTRASONIC PROCESSOR UP400ST machine at 0.5 pulse cycles and 50 % amplitude. This technique induces the breakage of CNT agglomerates due to the cavitation forces induced by the ultrasonic pulses. A study of the influence of sonication time on dispersion state was carried out, taking samples at initial state, 30 min, 1 h, 1.5 h, 2 h, and 2.5 h of sonication (Figure S1, Supplementary Materials). As indicated in the Supplementary Information, 2 h of this process was enough to guarantee a good CNT dispersion.

After the dispersion procedures, for both routes (3RM and US), a degassing step was carried out to evacuate the air trapped in the mixture during dispersion. For this, the mixture was heated to 80 °C to decrease its viscosity and then degassing was carried out in a rotatory pump under vacuum conditions (around 10^−5^ Pa) for 20 min on a magnetic stirrer. Then, PDMS curing agent was added in 10:1 proportion and manually mixed. Finally, the mixture was placed in the metallic mold previously smeared with a layer of release agent based on polyvinyl alcohol (CASTRO COMPOSITES^®^) and they were cured in an oven at 125 °C during 20 min, following the indications provided in the commercial PDMS data sheet.

### 2.3. Nanocomposite Characterization

#### 2.3.1. Microstructural Characterization

The CNT dispersion in the CNT-PDMS mixtures was analyzed by Light Transmitted Optical Microscopy (TOM), prior to the addition of the hardener and, therefore, before the curing step, to analyze the distribution of CNT immediately after the dispersion procedure. The microscope used was a Leica DMR equipped with a camera Nikon Coolpix 990.

Moreover, manufactured nanocomposites were cut with a microtomy blade, and their surfaces were coated with 3 nm of platinum using a sputtering Leica EM ACE200 machine. Surface samples were morphologically analyzed using Field-Emission Gun Scanning Electron Microscopy (FEG-SEM) Nova NanoSEM 230.

#### 2.3.2. Electrical Conductivity

**Figure 1:**
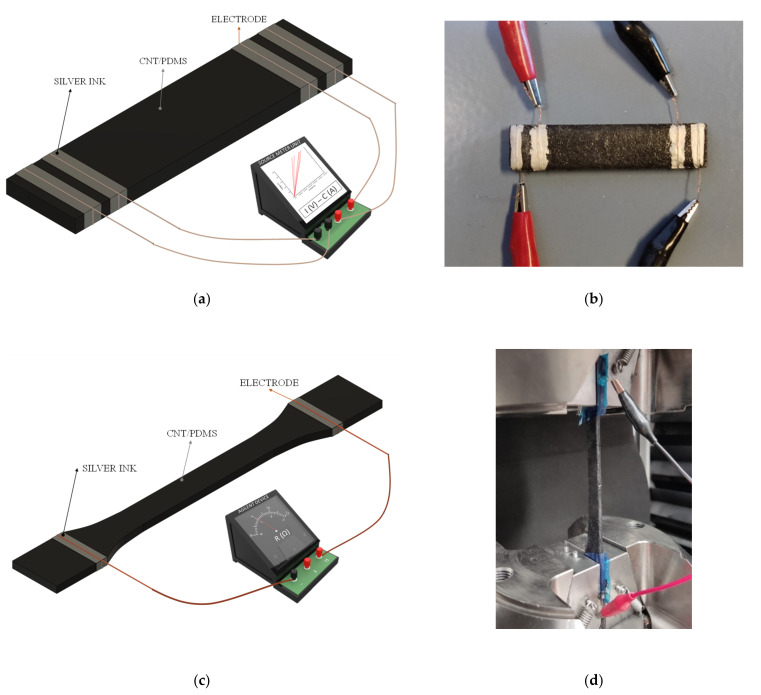
Scheme and real specimens for (**a**,**b**) electrical conductivity and (**c**,**d**) electromechanical tests.

The electrical volume conductivity was measured using a Source Meter Unit (Keithley Instrument Inc. mod. 2410). It was determined by calculating the slope of the current–voltage curve at 0–100 V for 0.25, 0.3, 0.4, and 0.5 wt.% CNTs and at 0–20 V for 0.75 and 1 wt.% CNTs, because of the higher electrical conductivity expected in the last ones. For these experiments, three specimens (60 × 20 × 6 mm^3^) were tested for each condition. Four electrodes made of copper wire were attached to the nanocomposite specimen using a conductive silver ink to make a four-probe measurement, with the aim of minimizing the contact resistance, as summarized in Figure 1a,b.

#### 2.3.3. Electromechanical Tests of CNT/PDMS Nanocomposites

The strain monitoring capabilities of nanocomposites were evaluated under tensile conditions using a ZWICK universal tensile machine equipped with a load cell of 500 N. At least three tensile specimens of each condition were tested according to ISO 527-1:2019, precisely, at a test rate of 10 mm/min. 

Synchronously to this mechanical test, the electrical resistance was measured using an Agilent 34410A module at an acquisition frequency of 10 Hz. For this purpose, two electrodes made of copper wire were placed around the nanocomposite surface near grips, approximately at 80 mm from the central region of the tensile specimen. These electrodes were attached to the nanocomposite using conductive silver ink to minimize the contact resistance, as shown in Figure 1c,d. Due to the hydrophobic nature of PDMS, it is important to note the difficulties related to electrode placement on the surface of nanocomposites. In addition, samples were isolated from the testing machine by applying an adhesive layer at the grips. 

A crucial parameter for characterizing the electromechanical capabilities of the proposed sensors is their Gauge Factor (*GF*), that can be defined as the electrical sensitivity to strain and can be calculated as the variation in the normalized electrical resistance (∆*R*/*R_0_*), divided by the applied strain (*ε*), as indicated in Equation (1):

(1) $$GF=\frac{\Delta R/R_{0}}{\varepsilon }$$

Additionally, to study the repeatability of the electrical response of the nanocomposite with the higher *GF*, which was the sample that contained 0.4 wt.% CNTs dispersed by the US procedure, three tensile specimens were subjected to continuous loading and releasing for 300 cycles up to 2%, 5%, and 10% strain level, at a fixed rate of 30 mm/min.

### 2.4. Proof of Concept

Finger and elbow movements were monitored as a proof of concept of the developed sensors, with the aim of proving their viability for human motion monitoring applications. For this reason, the nanocomposite with the higher GF (0.4 wt.% CNTs dispersed by US) was cut into strips of 20 × 8 × 1.5 and 60 × 20 × 1.5 mm^3^ dimensions and fixed on an index finger and on an elbow, respectively, using an adhesive layer. In addition, two copper wire electrodes were attached with silver ink to the ends of the samples to record the electrical response during the proof-of-concept test.

## 3. Results and Discussion

The electrical and electromechanical capabilities of CNT-PDMS were analyzed, exploring the influence of the dispersion procedure. Then, the applicability of the optimum sensor was proved under consecutive load cycling and with a human monitoring proof-of-concept such as the movements of elbows and fingers to characterize the sensitivity of the developed sensors.

### 3.1. Electrical Conductivity Measurements

**Figure 2:**
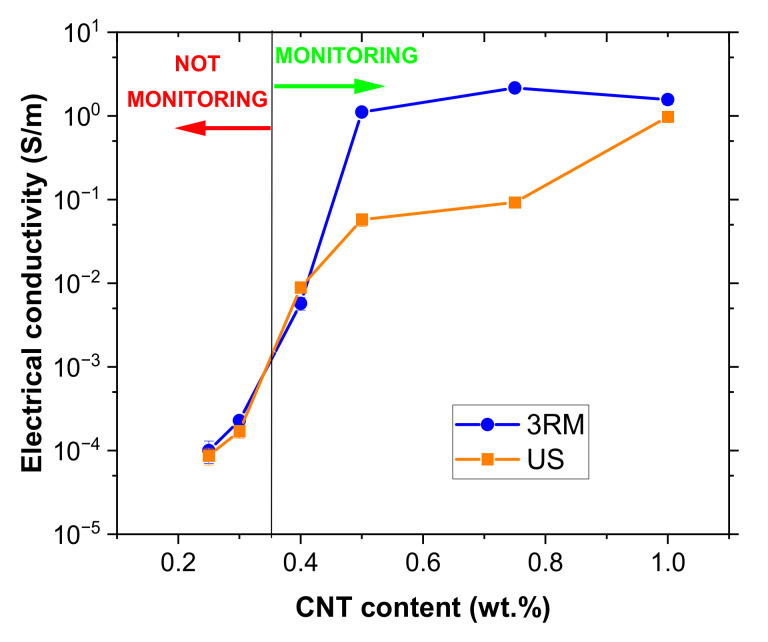
Electrical conductivity measurements for 3RM and US techniques as a function of CNT content.

Figure 2 shows the values of electrical conductivity measured for the different CNT contents tested for nanocomposites manufactured using the 3RM and US dispersion procedures.

First, it can be observed, as expected, that the electrical conductivity increases with the increasing CNT content due to the creation of more electrical pathways within the material, ranging from 10^−4^ at 0.25 wt.% to 3 S/m at 1 wt.%. Here, when comparing the effects of 3RM and US procedures on electrical properties, the following facts can be established.

At lower CNT contents, the electrical conductivity achieved by both dispersion routes is quite similar, reaching a percolation threshold below 0.25 wt.% in both cases. However, when the CNT content is increased above 0.4 wt.% the 3RM technique leads to significantly higher values of electrical conductivity than US. This behavior can be easily explained according to the higher efficacy of the first dispersion route.

In this regard, US is an effective dispersion procedure at low contents since the viscosity of the mixture is also low enough to make the cavitation forces induced during the sonication process more effective [30]. On the other hand, the 3RM procedure promotes the disaggregation of CNTs by the shear forces induced during milling. These shear forces are more prevalent when increasing the viscosity of the mixture [31], leading to a higher dispersion efficiency for higher CNT contents and, thus, promoting a significant increase in the electrical conductivity.

These statements about the influence of the dispersion procedure on the electrical properties of the nanocomposites were also confirmed by analyzing the CNT distribution achieved using Transmission Optical Microscopy (TOM) and Field-Emission Gun Scanning Electron Microscopy (FEG-SEM).

**Figure 3:**
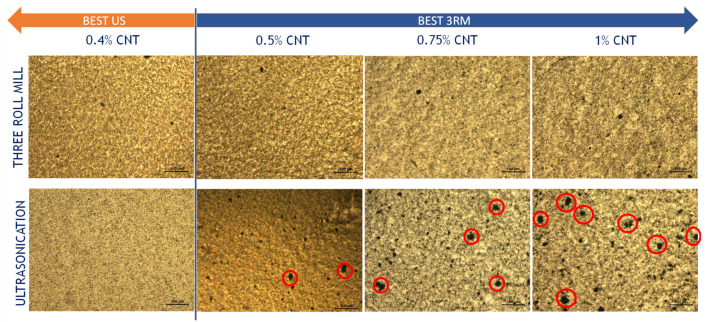
TOM images of the CNT-PDMS mixtures as a function of nanoparticle content and dispersion procedure. The areas highlighted in red denote the presence of larger CNT aggregates.

In this regard, Figure 3 summarizes a representative TOM image for each condition. It can be observed that, at lower contents (0.4 wt.%), the US dispersion process promotes a very homogeneous distribution due to the effectiveness discussed above of the cavitation forces in these low viscosity conditions. By increasing the CNT content (above 0.5 wt.%), the 3RM technique leads to much more homogeneous CNT distribution, and decreases, leading to the formation of larger aggregates (highlighted in red in Figure 3). Finally, it can be also stated that, at very high CNT contents (1 wt.%) there is no electrical conductivity in the nanocomposites processed by the 3RM route (there is even a slight decrease compared to the samples of 0.75 wt.% CNT). This can be explained because, although the higher viscosity of this more loaded mixture increases the shear forces, it also induces a breakage of CNTs themselves, leading to a detriment in the electrical properties. In fact, this effect has been widely reported in other studies, where this reduction in the aspect ratio of the nanoparticles has been observed, with the consequent loss of conductivity [32]. The influence of CNT breakdown mechanisms, however, is not as high for the US procedure as the effectiveness of this mechanism is reduced due to increased viscosity, as discussed above.

**Figure 4:**
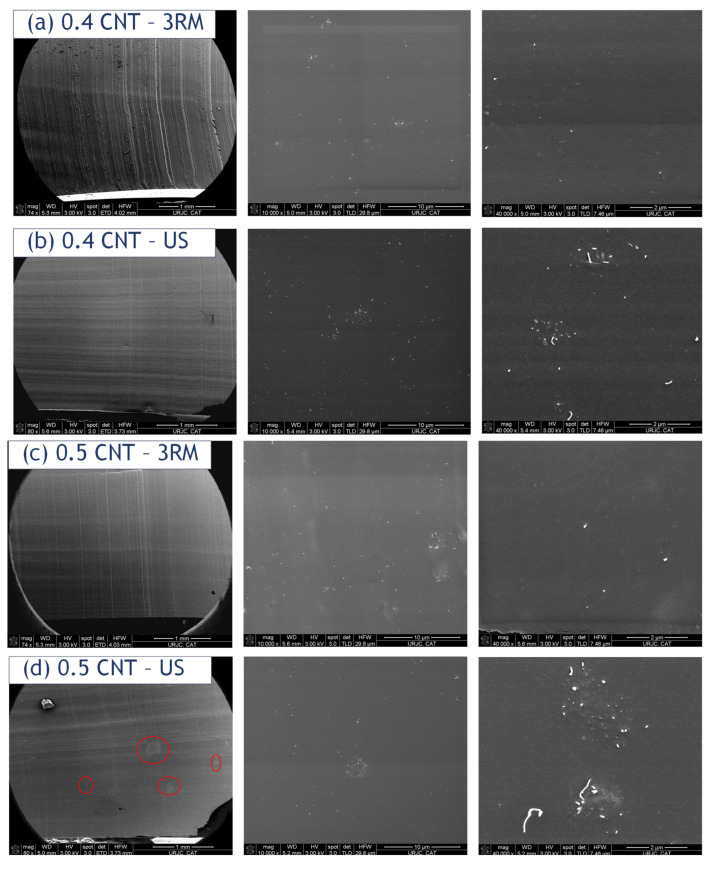

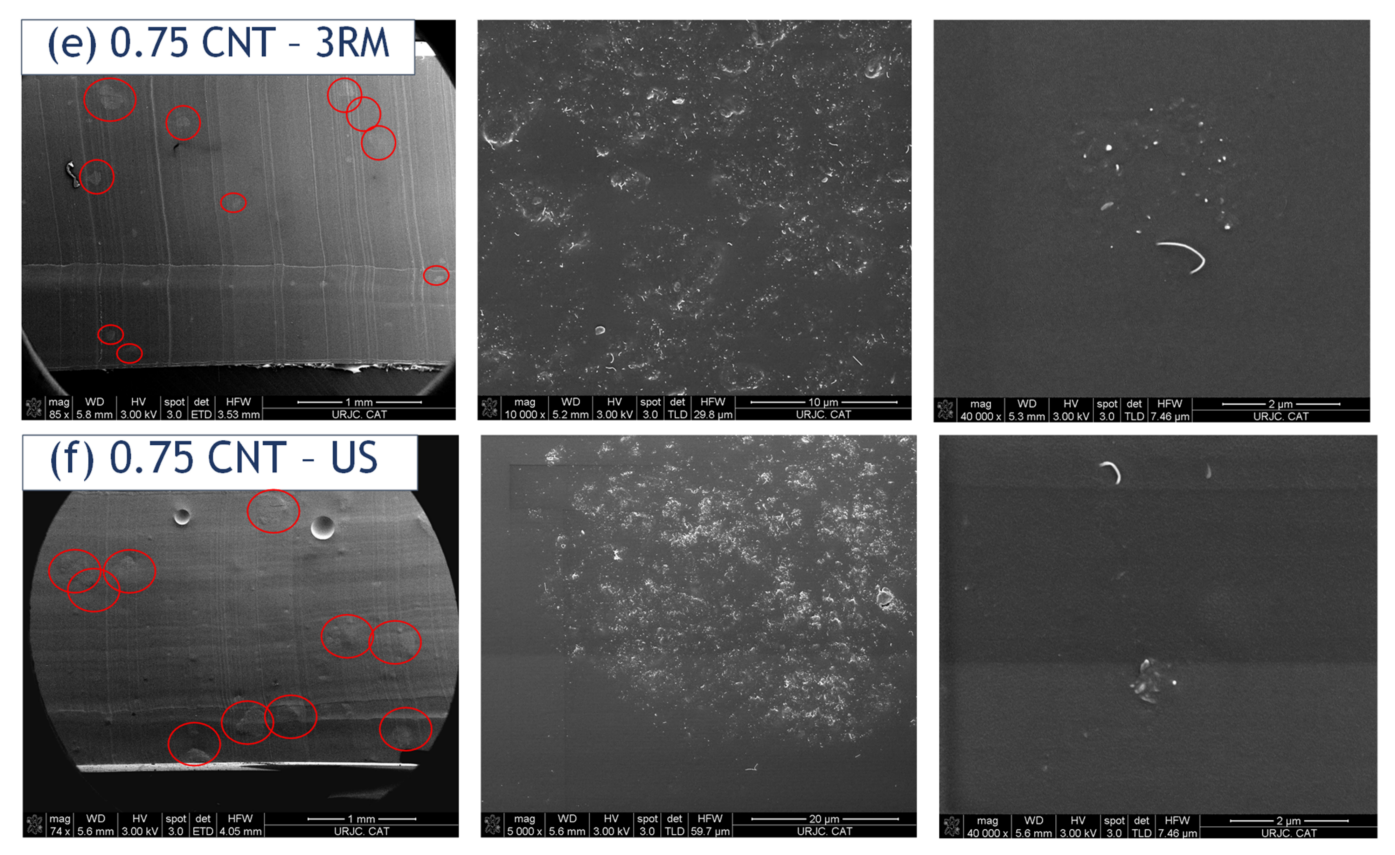
FEG-SEM images of the CNT-PDMS nanocomposites at (**a**,**b**) 0.4, (**c**,**d**) 0.5, and (**e**,**f**) 0.75 wt.% CNT as a function of dispersion procedure (US and 3RM). The left, center, and right images are at 70, 1000, and 40,000 magnifications, respectively. The areas highlighted in red denote the presence of larger CNT aggregates.

Figure 4 present FEG-SEM surface images at different magnifications. Here, the trend observed in TOM is corroborated, following the same arguments. On the one hand, nanocomposites with lower contents (0.4 wt.% shown in Figure 4a,b) promote a very homogeneous distribution with US and 3RM, with the absence of larger aggregates, and it is manifested in similar conductivity values with both techniques. On the other hand, nanocomposites with higher contents (0.5 and 0.75 wt.% shown in Figure 4c–f) present a better CNT dispersion with 3RM than US, because larger aggregates can be seen in US samples (especially at low magnifications). Therefore, higher conductivity values are achieved with this technique, as there are more efficient electrical pathways. It is important to highlight that no pull-out phenomena of the CNTs are observed at high magnifications in any of the samples, so there is a good interface between the PDMS and the CNTs.

### 3.2. Electromechanical Analysis

**Figure 5:**
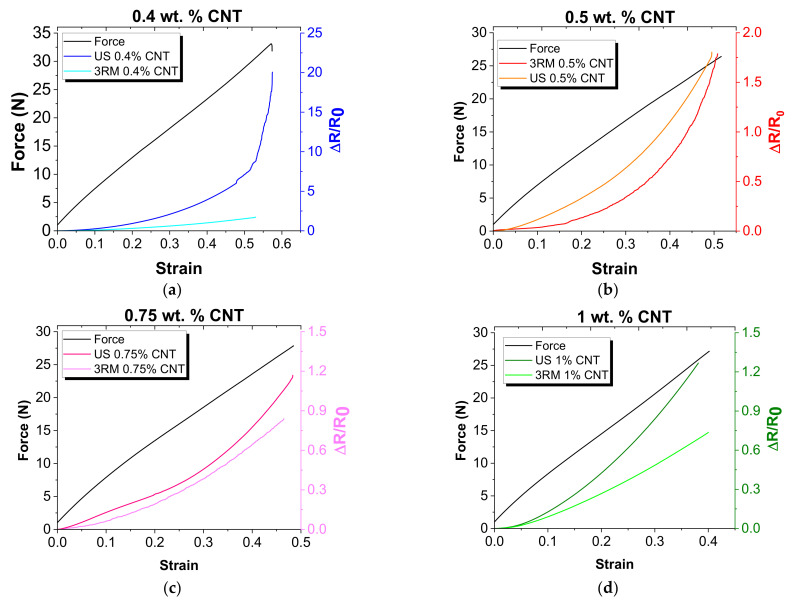
Strain monitoring curves of the CNT-PDMS sensors for the different manufacturing conditions and CNT contents: (**a**) 0.4 (**b**) 0.5, (**c**) 0.75, and (**d**) 1.0 wt.%.

Therefore, once the electrical properties of the CNT-PDMS nanocomposites have been analyzed following two different dispersion procedures, it is necessary to explore how they affect the strain sensing response of the developed sensors. In this context, Figure 5 summarizes some representative curves of the electromechanical behavior of the CNT-PDMS nanocomposites under tensile load.

First, it can be observed that only the conditions above 0.4 wt.% were successfully tested. This is explained because, although at lower contents the nanocomposites are electrically conductive, as observed in the graph of Figure 2, their conductivity was not high enough to be monitored since the electrical resistance was above the limits of the *Agilent* device.

When observing the electromechanical curves of Figure 5, it can be realized that the electrical resistance follows a linear-exponential behavior with the applied strain. This fact has been widely reported and it is correlated to the prevalence of tunnelling mechanisms in the electrical network formed in the nanocomposites that, according to the well-known Simmons formula [33], follows a linear-exponential trend with the distance between neighboring nanoparticles.

**Figure 6:**
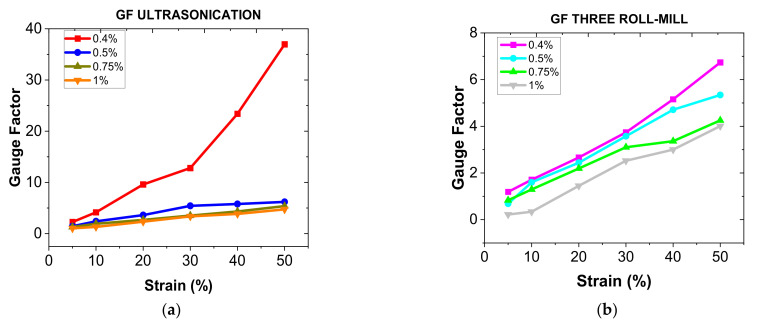
GF values obtained for (**a**) US and (**b**) 3RM CNT dispersion routes as a function of CNT content.

From these electromechanical curves, it is possible to estimate the nanocomposite’s GF, which is a key factor for strain sensing purposes. In this regard, Figure 6 shows the GF obtained as a function of CNT content for both 3RM and US following dispersion procedures.

Here, it can be observed that the GF increases with decreasing CNT content. It has been also widely reported and it is correlated to the previously mentioned prevalence of tunnelling mechanisms. In this regard, by increasing the CNT content and, thus, the number of nanoparticles, the average distance between particles decreases and, therefore, the exponential variation in the electrical resistance due to tunnelling mechanisms is less prevalent, leading, thus, to lower electrical sensitivities.

On the other hand, when comparing the dispersion procedures, it can be elucidated that the sensitivity is generally higher in nanocomposites processed following the US route, especially for lower CNT contents. This can be explained according to the dispersion state reached in the final nanocomposite.

In this regard, the US technique, as explained above, seems to be a very efficient dispersion method for lower CNT contents, due to the higher prevalence of cavitation forces during the dispersion stage. For this reason, the electrical network reached in the final nanocomposite is much more homogeneous and, thus, much more efficient, leading to a greater prevalence of tunnelling mechanisms, which present a very high sensitivity over the contact and intrinsic ones.

At higher contents, the 3RM process seems to be more efficient as a dispersion procedure so, at a first sight, the electrical sensitivity should be also higher than that obtained by ultrasonication technique. However, the significant differences between the electrical conductivity between the two dispersion routes suggests that the mean interparticle distance is much higher in the CNT network after the US procedure. Therefore, although there is also prevalence of conduction mechanisms through the CNT aggregates, this higher interparticle distance leads to an increase in the electrical resistance due to tunnelling mechanisms and, thus, to the electrical sensitivity of the samples.

The GF values, in every case, are much higher than those found in conventional metallic gauges for strain sensing purposes, which is around 2, especially when monitoring large strains. More specifically, the samples with 0.4 wt.% CNT processed following the US route have a GF of 3 and up to 37, at low and high strain levels, respectively. For this reason, this condition was selected as the optimal one for the development of wearable sensors since it also presents a very high failure strain (~50%).

**Figure 7:**
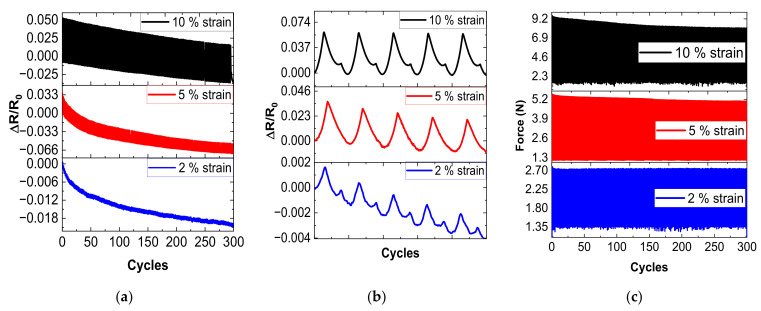
Cycling response of the 0.4 wt.% CNTs sensor by ultrasonication at 10%, 5%, and 2% strain levels. (**a**) Electrical response under 300 cycles; (**b**) detail of the first 5 cycles of the electrical response; and (**c**) mechanical response under 300 cycles.

In this regard, Figure 7 shows the electromechanical response under cycling load. The aim is to prove the repeatability of the electrical response and, thus, the robustness of the proposed sensors. As the sensors will be used for the monitoring of medium and large strains, such as elbow or finger movements, they were tested at a cycling strain of 2%, 5%, and 10% strain. Here, two significant facts can be stated: on the one hand, the sensitivity under cyclic load at medium-high levels is very high, reaching resistance changes up to 50% for 10% strain level, and increases with decreasing CNT content, as expected; on the other hand, the sensitivity of the reported sensors is constant over the entire range of cycles because the change in the electrical resistance (distance between the peak and baseline) remains almost constant for each consecutive cycle. However, the peak and baseline of the electrical resistance decrease with increasing numbers of cycles due to the viscoelastic behavior of PDMS that promotes a delayed mechanical response, as shown in Figure 7c, as well as some irreversibility in the electrical network that may be manifested, especially, in the first stages of the cyclic test. It denotes that the electrical network is fully recovered after applying the load, with the absence of microcracks or plastic deformation and proving, thus, demonstrating that the sensors can be used to reliably detect relatively small strains in a reliable way, without inducing any prevalent damage.

According to the state of the art on strain sensors, there are numerous research works focused on the development of wearable sensors based on CNT-reinforced silicon elastomers, especially with homemade silicone, Ecoflex, or PDMS. Table 2 summarizes the most prominent strain sensors developed in these investigations.

In this regard, in comparison with the sensitivity of the strain sensors in the reported studies, the results obtained in the present work provided significantly higher GF values than sensors manufactured with the similar CNT dispersion methods. It proves the importance of optimizing these dispersion procedures that was analyzed in the first sections of this work. On the other hand, although there are three developed sensors [14,20,23] that clearly exceed the GF values obtained in the present work, they were performed using much more complex and expensive manufacturing methods, such as CVD or laser ablation, than the one used in this study, which is the dispersion by ultrasonication.

The previous results show that the CNT-PDMS strain sensor developed in this research is in a privileged position with respect to the state of the art, considering all the characteristics that these types of sensors must meet, as was demonstrated in this study: high GF, high flexibility, high reproducibility, easy manufacturing, and friendly operation.

### 3.3. Proof of Concept: Human Motion Monitoring

**Figure 8:**
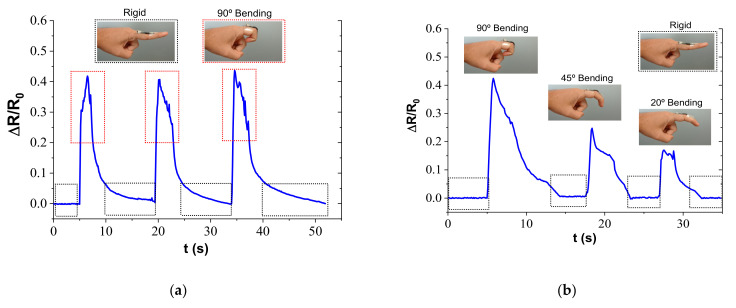

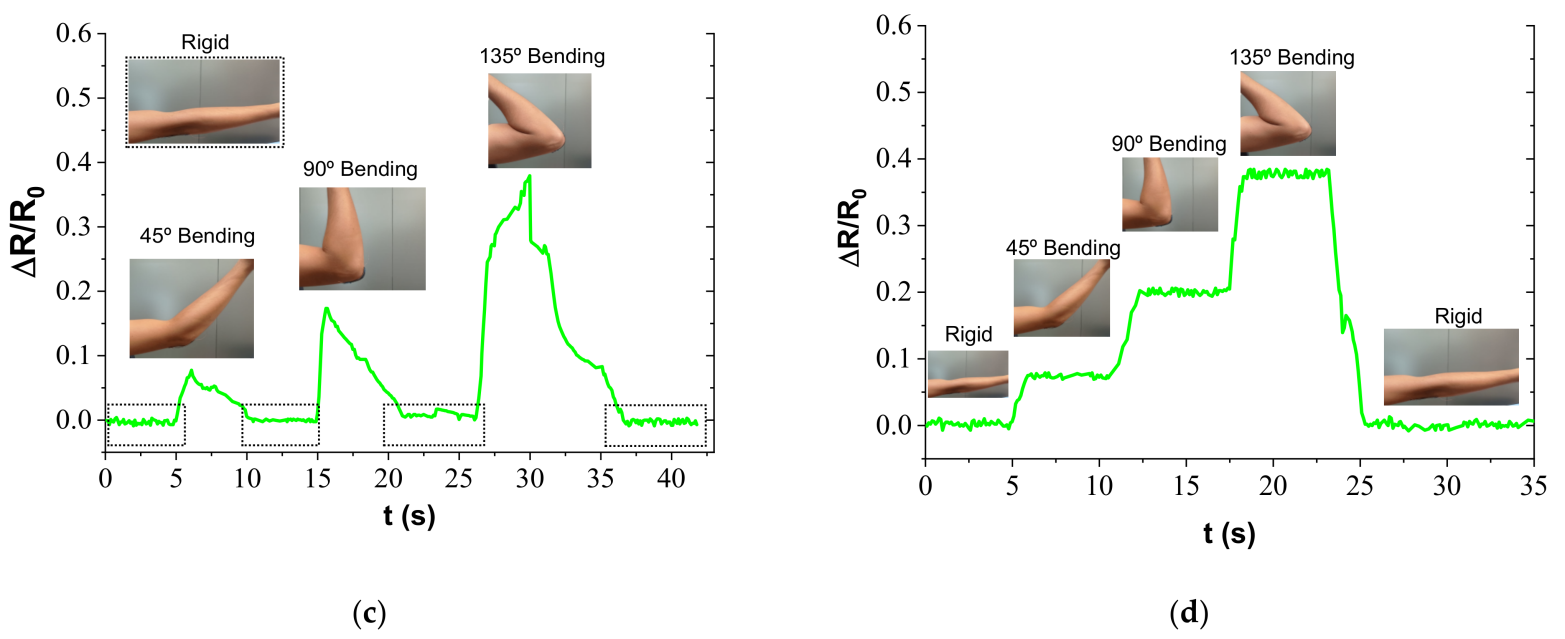
Electrical response to human motion monitoring when (**a**,**b**) finger and (**c**,**d**) elbow bending–holding.

The potential applications of the developed CNT-PDMS strain sensor for the detection of human body motions were investigated, as shown in Figure 8.

On the one hand, Figure 8a,b shows the monitoring of movement associated with flexion and extension of the index finger. In this regard, when the finger is flexed in the hand, a sudden increase in electrical resistance is detected due to the induced strain field in the attached sensor. This behavior can be explained by the extension of the CNT network, which is mainly subjected to tensile stresses. Subsequently, when the index finger is extended back to the initial position, the electrical resistance decreases to the initial value, due to the recovery of the initial configuration of the nanoparticle electrical network in the sensor PDMS matrix, as shown in Figure 8a. The recovery of the initial resistance denotes that the CNT sensor has not been severely damaged by the stresses generated by the movement of the finger, despite the great elongation associated with its bending. It denotes an adequate flexibility and resistance of the doped resin, responsible for ensuring the stability of the CNT electrical network. Furthermore, the CNT-PDMS sensor shows a response proportional between the level of flexion to which the finger is subjected and the increase in electrical resistance measured, showing great reproducibility and high sensitivity in detecting small movements of the monitored finger.

On the other hand, Figure 8c,d summarizes the electrical response under the bending and stretching movements of the arm. In a similar way as with the movements of the fingers, the greater the level of flexion of the arm, the greater the extension suffered by the network of nanotubes, increasing the tunnel distance between neighboring CNTs. The response to this extension of the network is the proportional increase in electrical resistance measured by the sensor. In every case, the electrical resistance recovers the initial value, indicating, again, that the sensors are not damaged during the elbow motion, even under the most demanding conditions (bending angle of 135°). In addition, Figure 8d shows the electrical response of the sensors when the arm is gradually bent, from an extended position to the maximum bending angle at 135°, ending with the arm being stretched. It can be observed that the electrical resistance increases with the bending angle, as shown previously, and the value remains stable as the bending angle is maintained. Finally, when the arm is stretched again, the electrical resistance returns to its initial value.

Consequently, the results of the proof-of-concept prove an outstanding robustness of the sensors for human motion monitoring and could open a number of applications of CNT-PDMS materials, manufactured in an easy and scalable way for these kinds of applications.

## 4. Conclusions

An easy-scalable flexible strain sensor based on CNT-reinforced PDMS was proposed for human motion monitoring.

The analysis of the dispersion procedure shows that the samples manufactured by the US technique show a higher electrical sensitivity than those manufactured by 3RM. This is explained by the homogeneous distribution achieved by US due to the prevalence of aggressive cavitation forces for the disaggregation of larger agglomerates, leading to a prevalence of tunnelling mechanisms. In addition, by increasing the CNT content there is a significant decrease in the sensitivity, as the interparticle distance is decreased due to the larger number of nanoparticles. In every case, the values of the gauge factor (3–37) clearly exceeded those found for metallic conventional gauges.

The analysis of the dispersion procedure shows that the samples manufactured by the US technique show a higher electrical sensitivity than those manufactured by 3RM. This is explained by the homogeneous distribution achieved by US due to the prevalence of aggressive cavitation forces for the disaggregation of larger agglomerates, leading to a prevalence of tunnelling mechanisms.

Based on this analysis, the optimum conditions for the manufacturing of the wearable sensors were selected as 0.4 wt.% CNT by means of an ultrasonication process. In this regard, an analysis of the electrical response under consecutive load cycles showed a high robustness of the developed sensors, without any presence of damage at 2, 5, and 10% strain, which make these sensors reliable for monitoring medium strain.

For these reasons, the optimized strain sensor is in a privileged position with respect to the state-of-the-art, considering all the characteristics that this type of sensor must meet: high GF, high flexibility, high reproducibility, easy manufacturing, and friendly operation

Finally, a proof-of-concept of human motion monitoring was carried out by placing a sensor on a finger and elbow. The electrical response during the bending and stretching of both the finger and elbow shows a reliable response with an increase in the electrical resistance with bending angle and a total recovery after stretching.

Therefore, these preliminary results show the huge potential of the proposed materials as wearable skin-mounted sensors for human motion monitoring as they are manufactured in an easy-scalable way and report a robust electrical behavior.

## Figures and Tables

**Table 1:** Process conditions for three roll milling dispersion at room temperature.

Cycle	First Roll Gap (μm)	Last Roll Gap (μm)
1	120	40
2	75	25
3	45	15
4–7	15	5

**Table 2:** The sensitivity and CNT dispersion/deposition method of strain sensors.

Silicone Matrix	CNT Dispersion or Deposition Method	Sensitivity (Gauge Factor)	Ref.
Silicone	US	0.99	[13]
Ecoflex	CVD	42,300	[14]
Ecoflex	Spray coating	3.5	[15]
Ecoflex	Spray coating	2	[16]
Ecoflex	Ultrasound bath	0.1–0.4	[17]
PDMS	CVD	0.97	[18]
PDMS	CVD	1.5	[19]
PDMS	CVD	100	[20]
PDMS	Spray coating	6.7	[21]
PDMS	Swelling/permeating	2–12	[22]
PDMS	Laser ablation	512.2	[23]
PDMS	Stirring manually	2.5	[24]
PDMS	Ultrasound bath	35.75	[25]
PDMS	US	0.98	[26]
*PDMS*	*US*	*3–37*	*This work*

## Data Availability

The data presented in this study are available on request from the corresponding authors.

## Supplementary Materials

The following supporting information can be downloaded at: https://www.mdpi.com/article/10.3390/s22145147/s1, Figure S1: Dispersion state of CNT/PDMS at different sonication times.
