# Supplementary material for: Easy-Scalable Flexible Sensors Made of Carbon Nanotube-Doped Polydimethylsiloxane: Analysis of Manufacturing Conditions and Proof of Concept
Source: Sensors (Basel). 2022 Jul 8;22(14):5147. doi: 10.3390/s22145147 (PMC9316376; doi:10.3390/s22145147)
Supplement: Supplementary file 1 [file sensors-22-05147-s001.zip › sensors-1794239-supplementary.pdf]

sensors

MDPI

SUPPLEMENTARY INFORMATION

Easy-scalable flexible sensors made of carbon nanotube-doped polydimethylsiloxane: Analysis of manufacturing conditions and proof of concept

Antonio del Bosque *, Xoan F. Sánchez-Romate, María Sánchez and Alejandro Ureña

Materials Science and Engineering Area, Escuela Superior de Ciencias Experimentales y Tecnología, Universidad Rey Juan Carlos, C/Tulipán s/n, 28933 Móstoles, Madrid, Spain; xoan.fernandez.sanchezromate@urjc.es (X.F.S.-R.); maria.sanchez@urjc.es (M.S.); alejandro.urena@urjc.es (A.U.)

* Correspondence: antonio.delbosque@urjc.es (A.d.B.), Tel: +34-914884621.

Figure S1 shows TOM images of the dispersion state at different sonication times for 0.5 wt.% CNTs mixtures. It can be observed that, at the initial stage, the CNTs are mainly aggregated (left and upper image). When increasing the sonication time, the agglomerates are effectively reduced, especially, when comparing 30 min, 1 h and 1.5 h of sonication time. However, larger sonication times such as 2.5 h do not promote a much better CNT dispersion inside the material (right and lower image). This fact can be easily explained by the effectiveness of sonication process, because of an increase in the sonication time might cause a detriment in the electromechanical properties due to a prevalence of breakage mechanisms of CNT themselves, without significantly improving the CNT dispersion. For this reason, 2 h sonication time is enough to guarantee the best CNT dispersion in this system.

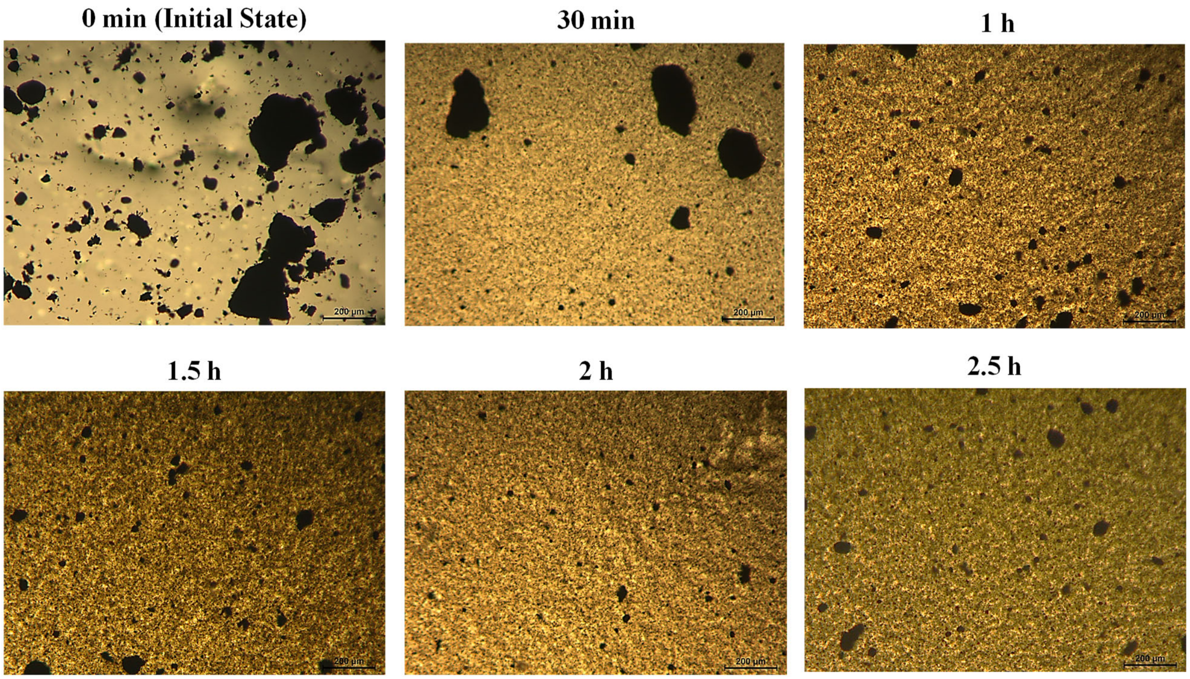

Figure S1: TOM images of the CNT/PDMS mixtures as a function of sonication time.
